# Robotic uterine transposition for fertility preservation in patients undergoing pelvic radiotherapy: a narrative review of surgical evolution, technical strategies, and emerging evidence

**DOI:** 10.1007/s11701-026-03296-7

**Published:** 2026-03-09

**Authors:** M. D’Indinosante, G. Parisi, Matteo Bruno, C. Innocenzi, M. Pavone, I. Peters, G. Corrado, D. Querleu, F. Fanfani, A. Fagotti, N. Bizzarri

**Affiliations:** 1 Dipartimento Scienze della Salute della Donna, del Bambino e di Sanità Pubblica, Unità Operativa Complessa di Ginecologia Oncologica, Fondazione Policlinico Universitario A. Gemelli, IRCCS, Largo Agostino Gemelli, 8, Rome, 00136 Italy; 2 Università Cattolica del Sacro Cuore, Largo Francesco Vito, 1, Rome, 00168 Italy; 3 Department of Woman’s and Child Health and Public Health Sciences, Ovarian Cancer Unit, Fondazione Policlinico Universitario A. Gemelli IRCCS, Largo A. Gemelli 8, Rome, 00168 Italy

**Keywords:** Uterine transposition, Robotic surgery, Minimally invasive surgery, Fertility preservation, Pelvic radiotherapy

## Abstract

Uterine transposition (UT) has emerged as a highly innovative fertility-preserving strategy for patients requiring pelvic radiotherapy. Unlike conventional approaches focused primarily on ovarian preservation, UT aims to protect both ovarian endocrine function and uterine integrity by temporarily relocating the uterus and adnexa outside the radiation field, followed by uterine repositioning after completion of oncologic treatment. While the feasibility of this procedure has been initially established through laparoscopic techniques, recent years have witnessed the introduction of robotic assistance, potentially offering advantages in surgical precision, visualization, and ergonomics during complex pelvic and upper abdominal dissection. This narrative review synthesizes the available evidence on robotic-assisted UT and contextualizes it within the previously published laparoscopic experience. The laparoscopic literature has defined patient selection criteria, operative principles, and early functional outcomes, demonstrating that UT can be safely integrated into multimodal oncologic treatment pathways. The emerging robotic experience, although limited to a small number of reported cases, confirms the technical feasibility of the procedure, with low perioperative outcomes and encouraging preservation of endocrine, reproductive and oncologic outcomes. Further evidence from prospective, multicenter experiences is required to define long-term outcomes and to clarify the role of robotic-assistance in this pioneering procedure.

## Introduction

Pelvic malignancies, including gynecologic, gastrointestinal, and genitourinary cancers, represent a substantial and growing global health burden, particularly among adolescents and young adults [1]. Recent epidemiological data indicate a progressive increase in the incidence of pelvic cancers diagnosed before the age of 40, driven by rising rates of colorectal cancer in younger populations and sustained incidence of cervical malignancies in reproductive-age women [2–4]. As multimodal oncologic strategies increasingly incorporate radiotherapy and chemotherapy to improve local control and survival, fertility preservation has emerged as a critical unmet need for young patients whose reproductive organs are not directly involved by the malignancy but are at risk of irreversible treatment-related damage. The increasing incidence of pelvic malignancies in these patients, combined with the progressive delay in childbearing age, has intensified the need for effective fertility-preserving strategies in oncologic care.

Pelvic radiotherapy remains a major threat to female reproductive potential. In patients whose uterus and ovaries are not directly involved by the malignancy, infertility risk arises predominantly from the collateral effects of radiation rather than from the disease itself [5].

Ovarian tissue is highly radiosensitive, with doses as low as 2 Gy resulting in substantial oocyte depletion, and age-dependent thresholds for permanent ovarian failure reported at approximately 20 Gy at birth, 18 Gy at 10 years, 16–17 Gy at 20 years, and 14 Gy at 30 years [6–8].

Beyond ovarian damage, radiotherapy exerts profound effects on uterine structure and function. Radiation could induce profound damage to the uterus, including myometrial fibrosis (which leads to reduced uterine volume and impaired distensibility), utero-vascular injury (with consequent altered perfusion), and endometrial receptivity dysfunction, ultimately compromising reproductive potential and obstetric outcomes. Clinical and imaging studies have consistently shown a dose-dependent reduction in uterine volume, increased uterine artery pulsatility indices, and loss of myometrial distensibility following pelvic irradiation. Notably, uterine fibrosis appears to correlate with cumulative radiation dose, with doses exceeding 45 Gy frequently associated with irreversible uterine damage. Importantly, adverse reproductive outcomes have been reported even at substantially lower uterine doses: exposure of the uterus to 2.5–5 Gy has been associated with significantly increased risks of miscarriage, fetal growth restriction, and low birth weight [9, 10].

These considerations underscore a limitation of conventional fertility-preservation strategies, which fail to protect uterine structure and function from radiation-related damage [11]. Consequently, in patients requiring pelvic radiotherapy without direct uterine involvement, strategies aimed at excluding the uterus from the radiation field have emerged as a logical extension of fertility-sparing care.

In this context, uterine transposition (UT) has emerged as a surgical option aimed at preserving both ovarian and uterine function by relocating the uterus and adnexa outside the radiation field, followed by anatomic uterine repositioning (UR) after completion of oncologic treatment [12–14]. By maintaining uterine vascular integrity and minimizing radiation exposure, this approach seeks to preserve not only hormonal function but also the structural and functional capacity of the uterus to sustain a future pregnancy.

While initial experiences established the feasibility of this approach through laparoscopy [13, 14], recent years have witnessed the increased use of robotic assistance, raising important questions regarding technical refinement, perioperative management, and the potential role of robotics in complex procedures.

The aim of this review was to describe the cases of UT performed by robotic assistance, to compare surgical technique and peri-operative outcomes of both approaches, and to critically appraise the emerging robotic experience within the context of previously published laparoscopic evidence.

## Methods

A comprehensive review of the literature was conducted to summarize current evidence on UT, with particular focus on the transition from laparoscopic to robotic-assisted approaches.

As no specific Medical Subject Headings (MeSH) term exists for UT, a broad search strategy was adopted. The primary search term *“uterine transposition”* was used to capture all relevant publications. Searches were performed in PubMed and Scopus, including articles published up to December 2025. Reference lists of key review articles were manually screened to identify additional eligible studies.

Inclusion criteria were: articles published in peer-reviewed scientific journals; studies describing clinical indications, surgical technique, perioperative management, outcomes, or oncologic considerations of UT. Exclusion criteria were: non-English language publications; non-clinical or experimental studies; articles lacking sufficient clinical or surgical detail relevant to UT. Conference abstracts, editorials, letters, and unpublished reports were excluded due to insufficient methodological detail and the inability to verify case duplication or clinical outcomes.

Titles and abstracts were screened for relevance, and eligible articles underwent full-text evaluation. Given the rarity of the procedure, the small number of reported cases, and heterogeneity in reporting, a formal systematic review or quantitative synthesis was not feasible. Therefore, findings were summarized using a descriptive narrative approach.

## Results

The literature search identified a limited body of evidence on UT, consisting primarily of laparoscopic case reports and small case series, largely synthesized in two comprehensive narrative reviews [13, 14]. Restricting the analysis to laparoscopic procedures only, a total of 18 UT cases have been reported, including 10 cases of rectal cancer [15–18], 4 cases of cervical cancer [19–21], and single cases of vaginal cancer [20], vulvar cancer [22], liposarcoma [17], and yolk sac tumor in a pre-pubertal patient [23]. Overall, seven individual robotic UT cases were identified and included in the present analysis (Table 1). These cases, all published as video-articles, form the basis for the descriptive assessment of patient characteristics, surgical strategies, and early outcomes associated with robotic-assisted UT. Owing to the small sample size, heterogeneity in reporting, and absence of comparative cohorts, no formal quantitative meta-analysis was performed.

The mean age of patients undergoing robotic UT was 30 years (range 23–36 years). Rectal adenocarcinoma represented the most frequent indication for UT, accounting for 4 of 7 cases (57%), followed by two cases of cervical carcinoma (28%) and one case of vaginal squamous cell carcinoma (15%). One patient presented with an additional anatomical complexity due to the presence of a sizable intramural uterine fibroid.

Disease staging reflected locally advanced but non-metastatic disease, with nodal involvement described in 6 cases (86%). All patients required pelvic radiotherapy as part of their oncologic management. Follow-up duration was variably reported across studies, with a median follow-up of 14 months (range 4–26 months).

**Table 1:** Clinical characteristics of reported robotic-assisted uterine transposition cases

Case ID	Reference	Age (years)	Primary tumor	Stage	Medical treatment	Surgical approach	Follow-up after UR (months)
**UT-01**	Marques et al. [24]	28	Cervical squamous cell carcinoma	FIGO IIIC1	Adjuvant chemoradiotherapy (pelvic EBRT 45 Gy)	Robotic-assisted (Da Vinci Si)	20
**UT-02**	Marques et al. [25]	30	Cervical adenocarcinoma, Silva type C	FIGO IIIC1	Adjuvant chemoradiotherapy (pelvic EBRT 45 Gy)	Robotic-assisted (Da Vinci Si)	26
**UT-03**	Gornet et al. [26]	36	Rectal adenocarcinoma with uterine intramural fibroids	cT3N2M0	Neoadjuvant chemoradiotherapy (pelvic RT)	Robotic-assisted	4
**UT-04**	Leitao et al. [27 28]	27	Rectal adenocarcinoma	cT2N+M0	Neoadjuvant chemoradiotherapy (pelvic RT)	Robotic-assisted	12
**UT-05**	Guijarro-Campillo et al. [29]	30	Vaginal squamous cell carcinoma	FIGO III	Neoadjuvant chemoradiotherapy	Robotic-assisted	NR
**UT-06**	Hsiao et al. [30]	23	Rectal adenocarcinoma	AJCC IIIA cT2aN1M0	Noadjuvant chemoradiotherapy	Robotic-assisted (Da Vinci Si)	12
**UT-07**	Bizzarri et al. [31]	35	Rectal adenocarcinoma	cT4N0M0	Neoadjuvant chemoradiotherapy (pelvic EBRT 60.1 Gy) followed by consolidation chemotherapy	Robotic-assisted (Da Vinci Xi)	12

Leitao et al. 2024a reports the robotic-assisted uterine transposition step, while Leitao et al. 2024b describes the subsequent uterine repositioning and anastomosis performed in the same patient. *Abbreviations: CRT = chemoradiotherapy; NR = not reported; RT = radiotherapy; UR = uterine repositioning; UT = uterine transposition*

## Evolution of uterine transposition and rationale for robotic assistance

### Laparoscopic description

UT was first introduced in 2015 by Ribeiro et al. as a fertility-preserving surgical strategy for a 26-year-old patient with locally advanced rectal adenocarcinoma undergoing neoadjuvant treatment and pelvic irradiation, providing the first proof of concept that complete transposition of the uterus, ovaries, and fallopian tubes outside the radiation field could preserve both endocrine and uterine function [15] .

Following this initial report, subsequent laparoscopic experiences progressively expanded the clinical application of UT and clarified its technical boundaries.

Across these early experiences, several core technical principles progressively emerged and were consistently adopted. These included extensive mobilization and skeletonization of the infundibulo-pelvic ligaments to enable tension-free cranial transposition, cautious and low transection of the uterine vessels to preserve collateral blood supply, and individualized strategies for cervical management aimed at minimizing ischemic risk. Together, these concepts established the technical foundation of laparoscopic UT and informed subsequent refinements in surgical technique [13, 14].

The laparoscopic approach also highlighted the inherent technical complexity of the procedure. Dense pelvic anatomy, prior pelvic surgery, and the frequent need for coordination with colorectal or radiation oncology teams posed challenges in terms of exposure, ergonomics, and precision.

### Patient selection and indications

Formal patient selection criteria for UT were initially standardized within a prospective multicenter study and subsequently adopted as the reference framework in later reviews [17]. Eligible candidates were women aged leq 40 years with a strong desire for future pregnancy, who required pelvic radiotherapy and demonstrated normal baseline uterine and ovarian function.

Oncologic and anatomical exclusion criteria included: tumor infiltration of reproductive organs, indication for para-aortic radiotherapy, prior pelvic/para-aortic irradiation, or metastatic disease; uterine volume exceeding 300 mL, prior surgeries compromising ovarian vascular pedicles and a history of infertility. Crucially, UT must be managed within a multidisciplinary framework and must never delay or modify the standard oncologic treatment plan. Following UT treatment, UR was typically planned 2–4 weeks after radiotherapy completion.

### Rationale for robotic assistance

The introduction of robotic assistance in UT should be interpreted within the broader evolution of minimally invasive surgery. Over the last two decades, robotic platforms have progressively gained diffusion in gynecologic and pelvic surgery, driven by technological maturation, increasing availability of competing systems, and a gradual reduction in costs, leading to wider adoption across high-volume referral centres [32]. Robotic surgery has been shown to facilitate complex pelvic and abdominopelvic procedures by providing stable three-dimensional visualization, articulated instruments with enhanced dexterity, tremor filtration, and improved ergonomics, particularly in operations requiring prolonged dissection, precise vascular preservation, and advanced suturing in confined anatomical spaces [33]. These characteristics are especially relevant in UT, a technically demanding procedure that combines extensive cranial mobilization of the gonadal vessels, meticulous handling of uterine perfusion, and complex fixation or anastomotic steps.

Emerging robotic-assisted experiences in UT have consistently adhered to the same patient selection framework established by laparoscopic series, without redefining oncologic indications. Rather than expanding eligibility, robotic assistance has been applied in technically more demanding clinical contexts, where procedural complexity arises from anatomical factors, treatment sequencing, or the need for multidisciplinary surgical integration.

Within this evolutionary framework, Moretti-Marques et al. first reported the use of a robotic platform (da Vinci Si, Intuitive Surgical) for UT in 2020, demonstrating the technical feasibility of reproducing the laparoscopic principles of the procedure using robotic assistance [24]. Subsequently, the same group reported the first spontaneous pregnancy and live birth following robotic UT, providing preliminary obstetric evidence after robotic-assisted transposition and repositioning [25].

Multidisciplinary evaluation remains central to this approach, with close coordination among gynecologic oncologists, colorectal surgeons, radiation oncologists, fertility specialists, radiologists, and supportive care teams to ensure appropriate patient counseling and operative planning.

## Surgical technique and perioperative outcomes

Reporting of surgical metrics was heterogeneous across articles, reflecting the descriptive nature of the available evidence and precluding formal quantitative synthesis. Across the reported robotic-assisted UT cases, the surgical strategy closely mirrors the laparoscopic principles established in earlier series, with adaptations enabled by robotic technology. In all cases, UT was performed prior to pelvic radiotherapy and consisted of four essential phases: (a) standardized trocar positioning and robotic docking tailored to combined pelvic and upper abdominal access; (b) pelvic and retroperitoneal access with systematic exposure and mobilization of the uterus, adnexa, and gonadal vessels; (c) cranial UT with fixation of the uterus and adnexa to the anterior abdominal wall; (d) and delayed UR and cervico-vaginal reconstruction following completion of oncologic treatment.

### A. Trocar positioning and docking strategy

**Fig. 1:**
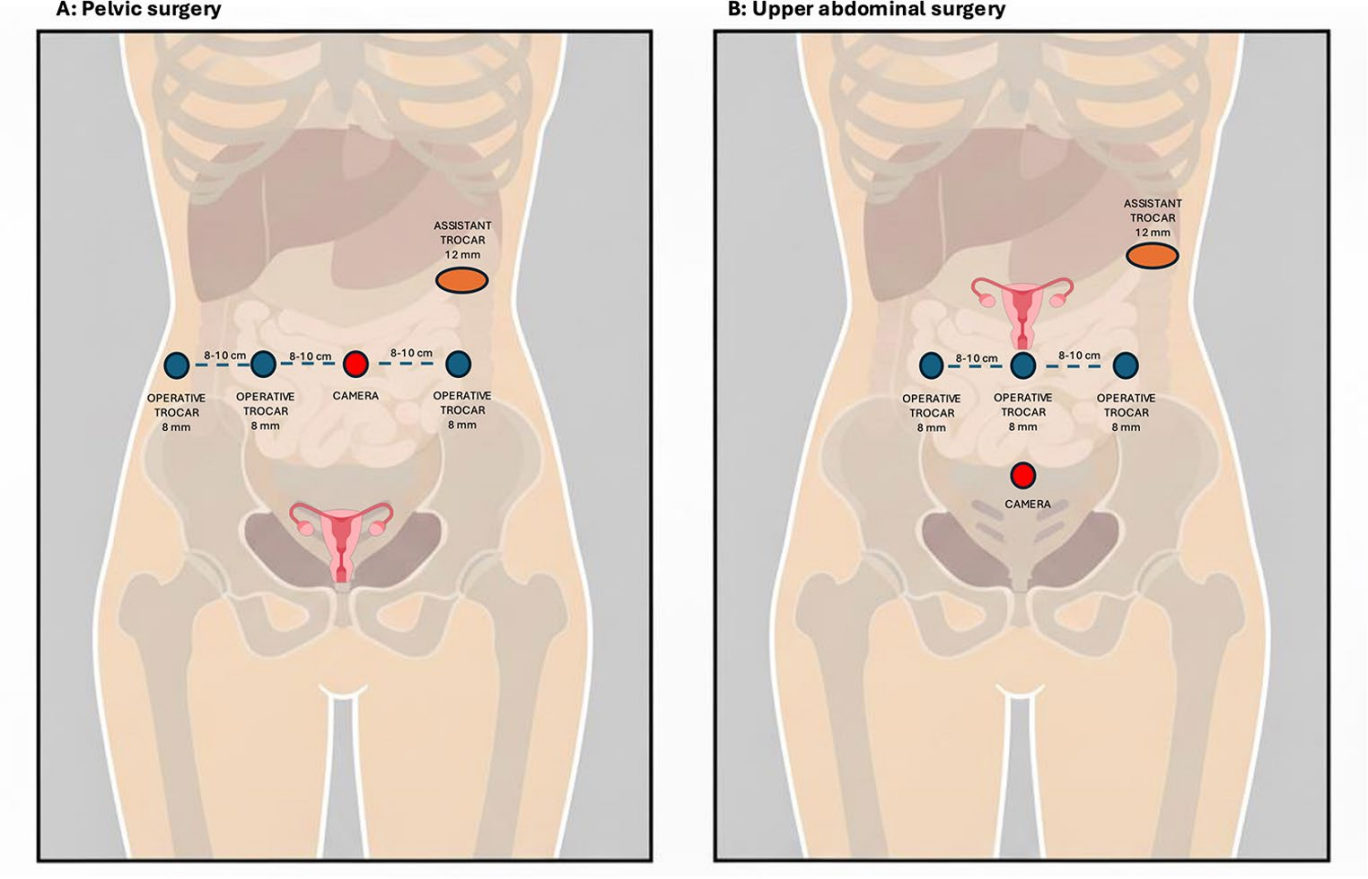
Schematic representation of trocar positioning for the dual-phase uterine transposition procedure

Across reported robotic cases, trocar positioning was planned as a two-phase procedure, consisting of an initial pelvic dissection followed by cranial mobilization and fixation of the uterus in the upper abdomen (Fig. 1).

In the pelvic phase, most authors adopted a trocar layout comparable to that used for robotic hysterectomy, with the camera port placed infra or supra-umbilically and working ports distributed laterally to allow safe retroperitoneal dissection and precise handling of the uterine vessels. The authors emphasize the importance of adequate spacing (8–10 cm) between robotic ports to avoid arm collision during extensive parametrial and infundibulo-pelvic ligament dissection, particularly in patients with prior pelvic surgery or distorted anatomy [27].

A distinctive feature of robotic UT is the planned reconfiguration of ports and docking for the upper abdominal phase. Leitao et al. describe a strategy in which a suprapubic trocar placed during the pelvic phase is later reassigned as the camera port for cranial dissection, without the need for additional trocar placement at that step. This approach allows optimal visualization of the gonadal vessels as they course toward the para-aortic region and facilitates extensive cranial skeletonization of the infundibulo-pelvic ligaments while maintaining ergonomic instrument angles [27]. 

Guijarro-Campillo et al. similarly stress the importance of positioning the camera as low as possible during the pelvic phase, with a 30-degree upward orientation, to optimize visualization during subsequent cranial transposition and fixation. The use of a 12-mm assistant port was consistently reported as advantageous during the abdominal phase, allowing repeated introduction and retrieval of sutures during fixation of the uterus, cervix, ovaries, and round ligaments to the anterior abdominal wall [29].

### B. Access, exposure and mobilization

The procedure typically begins with systematic bilateral retroperitoneal access and extensive skeletonization of the infundibulo-pelvic ligaments, performed cranially well above the pelvic brim. This step represents a cornerstone of UT, consistently emphasized as critical to achieve tension-free cranial mobilization of the uterus and ovaries, thereby minimizing the risk of vascular compromise. Excessive tension on these structures has been implicated in compromised perfusion and rare cases of uterine necrosis in the laparoscopic series. Robotic assistance facilitates this phase through enhanced three-dimensional visualization and articulated instrumentation, allowing precise dissection around the ureter and gonadal vessels even in patients with prior pelvic surgery or distorted anatomy.

Mobilization of the sigmoid or descending colon is performed along the avascular plane of the white line of Toldt. Extensive ureterolysis is emphasized across series to ensure constant visualization throughout the procedure.

After lateralization of the ureter, the round ligaments are divided laterally, preserving adequate length to allow subsequent tension-free fixation to the abdominal wall.The posterior leaf of the broad ligament is then incised to access the uterine vascular pedicles. The uterine vessels are skeletonized, sealed and cut, lateral to the cervix, with particular attention to minimizing thermal spread in order to preserve cervical perfusion, and as low as possible to preserve as much of the collateral blood supply to the cervix/uterus.

The anterior leaf of the broad ligament was opened bilaterally, followed by incision of the vesical fold and the dissection of the vesicouterine space. Retrocervical peritoneum is incised and dissected.

Colpotomy is then performed, typically with the aid of an atraumatic uterine manipulator to optimize exposure while minimizing direct handling of the uterine corpus and cervix. Colpotomy was typically performed using cutting mode energy to limit thermal injury to the vaginal cuff. The vaginal cuff is closed with absorbable sutures, taking into account the anticipated exposure to pelvic radiotherapy and the associated risk of impaired healing.

### C. Uterine Transposition (UT)

Surgical technique and perioperative outcomes of robotic-assisted UT are summarized in Table 2.

Pharmacologic menstrual suppression was employed in six robotic cases, allowing avoidance of menstruation during the transposed phase.

Cervical management represents a key technical variable. Unlike early laparoscopic experiences, in which the cervix was intentionally exteriorized through the umbilicus, robotic series have consistently favored intra-abdominal cervical fixation in six of seven cases (86%), with umbilical cervical exteriorization reported in only one case (14%) [30].

Subsequent cranial transposition of the uterus was achieved by relocating the organ to the anterior upper abdominal wall, most commonly at or slightly above the level of the umbilicus, ensuring tension-free positioning of the infundibulopelvic ligaments. Fixation was typically achieved in a stepwise fashion, beginning with the round ligaments, followed by the uterine fundus and ovaries, using absorbable barbed sutures. Particular attention was paid to avoiding torsion of the infundibulo-pelvic ligaments and to maintaining a physiological orientation of the adnexa. At this point pneumoperitoneum insufflation was reduced by some authors to 8–10 mmHg to minimize tension on the infundibulopelvic ligaments.

Operative time for UT was explicitly reported in a single case, with a duration of 240 min [31]. In the remaining cases, operative time was not specified. When reported, estimated blood loss during UT was minimal [28], with no case documenting major blood loss. The median length of hospital stay after UT was 2 days (ranging from same-day discharge to 3 days).

**Table 2:** Surgical technique and perioperative outcomes of robotic-assisted uterine transposition procedures

Case ID	Menstrual suppression	Cervical position during UT	UT operative time (min)	UT EBL (mL)	UT LOS (days)
**UT-01**	NR	Intra-abdominal fixation	NR	NR	NR
**UT-02**	Continuous combined oral contraceptives	Intra-abdominal fixation	NR	NR	3
**UT-03**	GnRH agonist	Intra-abdominal fixation	NR	NR	1
**UT-04**	GnRH agonist	Intra-abdominal fixation	NR	NR	1
**UT-05**	GnRH agonist	Intra-abdominal fixation	NR	NR	NR
**UT-06**	GnRH agonist	Umbilical exteriorization	NR	NR	NR
**UT-07**	GnRH agonist	Intra-abdominal fixation	240	Minimal	3

*Abbreviations: EBL = estimated blood loss; LOS = length of stay; NR = not reported; UT = uterine transposition*

### D. Uterine Repositioning (UR)

Timing, associated procedures, and perioperative outcomes of UR following robotic-assisted uterine transposition are summarized in Table 3.

UR was performed either as a staged procedure following completion of pelvic radiotherapy or synchronously with definitive oncologic surgery, depending on the underlying treatment strategy and multidisciplinary planning. In reported cases, UR was combined with additional oncologic procedures, including low anterior resection, bilateral inguinal-femoral lymphadenectomy, and transanal local excision [28–30].

Both laparoscopic and robotic approaches were adopted for the repositioning phase, reflecting institutional expertise and operative context.

This phase consistently involved adhesiolysis of the transposed uterus and adnexa, release of fixation sutures from the anterior abdominal wall, and restoration of pelvic anatomy. Particular attention was paid to the infundibulopelvic ligaments and utero-ovarian pedicles, which had been previously extensively mobilized, to avoid torsion or undue traction during descent of the uterus into the pelvis. Re-approximation of the round ligaments was routinely performed to provide additional uterine support and restore physiological orientation.

Cervico-vaginal anastomosis was typically performed via a transvaginal approach using running or interrupted absorbable sutures. This approach allowed direct intraoperative assessment and tailoring of the cervical length protruding into the vaginal canal, facilitating optimal anatomical restoration and potentially reducing the risk of postoperative cervical stenosis, dyspareunia, or functional mismatch.

Operative time for UR varied according to surgical context. When UR was performed as a stand-alone procedure, operative time was 165 min [31], whereas combined procedures involving synchronous colorectal surgery required longer operative time up to 300 min [27, 28]. The only quantified blood loss (60 mL) was reported during UR in one patient undergoing synchronous low anterior resection [27, 28]. The median length of hospital stay after UR was 3 days (range 2–4 days).

**Table 3:** Timing, associated procedures, and perioperative outcomes of uterine repositioning procedures

Case ID	UR timing	UR associated surgery	UR operative time (min)	UR EBL (mL)	UR LOS (days)
**UT-01**	NR	None	NR	NR	NR
**UT-02**	20 days after medical treatment	None	NR	NR	2
**UT-03**	7 months after UT and 2 month after medical treatment	None	NR	NR	NR
**UT-04**	NR	Lower anterior resection	300	60	4
**UT-05**	NR	Bilateral inguinal femoral lymphadenectomy	NR	NR	NR
**UT-06**	NR	Transanal local excision	NR	NR	NR
**UT-07**	8 months after UT and 1 month after transanal local excision	None (transanal local excision previously performed)	165	Minimal	3

*Abbreviations: EBL = estimated blood loss; LOS = length of stay; NR = not reported; UR = uterine repositioning; UT = uterine transposition*

#### Perioperative and postoperative assessment and complications

Perioperative and postoperative functional assessment, imaging, and reported complications related to uterine transposition and uterine repositioning are detailed in Table 4.

Assessment of uterine perfusion using indocyanine green (ICG) fluorescence imaging was reported in six robotic cases [25–31]. Although dosing protocols were inconsistently detailed, intraoperative intravenous ICG consistently demonstrated preserved perfusion of the uterine fundus and adnexa following transposition. Robotic near-infrared imaging allows real-time visualization of uterine and ovarian blood flow and may represent an additional safety measure in this context.

In two cases (28%) tubal patency was confirmed using methylene blue chromopertubation, highlighting the potential role of this functional testing at the end of anatomic restoration [26, 31] .

Ultrasound assessment was reported in five cases (71%) and was performed at variable time points following uterine transposition and/or subsequent uterine repositioning (range 1–7 days).

Postoperative complications were uncommon but clinically relevant. One patient developed postoperative small bowel obstruction three weeks after UT, requiring re-laparoscopy (Clavien–Dindo grade IIIb) [26]. Two patients experienced late cervical stenosis, necessitating surgical cervical dilatation after UR (Clavien–Dindo grade IIIa) [24, 25]. No intraoperative complications directly attributable to the transposition or repositioning procedures were reported. No cases of uterine necrosis, irreversible ischemia, or acute vascular compromise were observed across the published robotic series.

**Table 4:** Perioperative and postoperative functional assessment, imaging, and reported complications related to uterine transposition and subsequent uterine repositioning

Case ID	ICG IV injection	Tubal patency testing	US assessment	Complications
**UT-01**	NR	NR	NR	Late cervical stenosis requiring cervical dilatation 12 months after UR
**UT-02**	1 ml (25 mg/10 ml) during UT	NR	After UT (no timing reported)	Late cervical stenosis requiring cervical dilatation 6 months after UR
**UT-03**	During UT	Yes (methylene blue chromopertubation)	1 day after UT	Small bowel obstruction requiring re-laparoscopy 3 weeks after UT
**UT-04**	4 ml (25 mg/10 ml) during UT and UR	NR	1 week after UT and UR	None
**UT-05**	During UT	NR	After UT (no timing reported)	None
**UT-06**	1 ml (25 mg/10 ml) during UT and UR	NR	NR	None
**UT-07**	During UT and UR	Yes (methylene blue chromopertubation)	After UT and UR (no timing reported)	None

*Abbreviations: ICG = indocyanine green; IV = intravenous; NR = not reported; UR = uterine repositioning; US = ultrasound; UT = uterine transposition*

## Endocrine, fertility and oncologic outcomes

Endocrine, fertility, and oncologic outcomes following robotic-assisted UT and subsequent UR are summarized in Table 5.

**Table 5:** Endocrine, fertility, and oncologic outcomes after robotic-assisted uterine transposition and repositioning

Case ID	Return of menses	Endocrine outcome	Fertility outcome	Oncologic outcome	ΔUT to RT (days)	Follow-up (months)
**UT-01**	Yes	Normal hormonal function reported	Not attempted	No evidence of disease	NR	20
**UT-02**	Yes	Normal hormonal function reported	Spontaneous pregnancy 12 months after UR resulting in live birth after cesarian section	No evidence of disease	NR	26
**UT-03**	Yes (at 3 months after UR)	Normal hormonal function reported	NR	No evidence of disease	15	4
**UT-04**	Yes (2 months after GnRH discontinuation)	FSH and estradiol in premenopausal range	NR	No evidence of disease	28	12
**UT-05**	NR	NR	NR	No evidence of disease	14	NR
**UT-06**	Yes	AMH within normal range at 11 months after UR	NR	No evidence of disease	NR	12
**UT-07**	Yes	Normal hormonal function reported	Not attempted	No evidence of disease	14	12

*Abbreviations: NR = not reported; RT = radiotherapy; UR = uterine repositioning; UT = uterine transposition*

### Endocrine outcomes

Preservation of ovarian endocrine function was consistently reported across the robotic-assisted UT cases. Five patients (71%) received pharmacologic ovarian suppression with gonadotropin-releasing hormone (GnRH) analogues during oncologic treatment, while one patient (14%) was managed with continuous combined oral contraceptives [25].

Return of spontaneous menstruation was documented in six (86%) patients after UR and discontinuation of hormonal suppression. When reported, postoperative hormonal profiles confirmed preserved ovarian function, with follicle-stimulating hormone and estradiol levels remaining within the premenopausal range. In one case, anti-Müllerian hormone levels measured 11 months after UR were within normal limits, further supporting preservation of ovarian reserve [30]. No cases of permanent ovarian failure or irreversible endocrine dysfunction were described in the robotic series.

### Fertility outcomes

Data on fertility and obstetric outcomes following robotic-assisted UT remain limited by short follow-up and the small number of reported cases.

One patient was reported to attempt conception, resulting in a spontaneous pregnancy and live birth, following robotic UT and subsequent UR, and describing a successful singleton pregnancy carried to the late preterm period by cesarean Sect [25]. Two patients (28%) were reported not to have attempted pregnancy yet [24, 31]. In the remaining cases, patients had not yet attempted conception at the time of publication.

### Oncologic outcomes

Oncologic outcomes following robotic-assisted UT were reassuring within the limits of available follow-up. At last reported follow-up, no local pelvic recurrences or distant metastases were observed [24–31].

All patients (7/7) completed the planned oncologic treatment, including pelvic radiotherapy, without modification attributable to the UT procedure. In the four robotic cases in which the interval between UT and the start of oncologic treatment was explicitly reported, radiotherapy was initiated after a mean of 18 days (range 14–28 days). Importantly, UT did not interfere with radiotherapy delivery, surgical margins, or oncologic decision-making in any reported case.

## Discussion

### Summary of main findings

The present narrative review synthesizes the available evidence on robotic-assisted UT and contextualizes it within the established laparoscopic experience. The laparoscopic literature, encompassing 18 unique cases reported to date, has laid the technical and conceptual foundations of UT, defining patient selection criteria, operative principles, and early functional and oncologic outcomes [13–23]. These experiences demonstrated that UT can be integrated into multimodal oncologic treatment pathways without compromising cancer management, provided that patients are carefully selected and managed within a multidisciplinary framework.

The emerging robotic experience, currently limited to seven reported cases [24–31], does not redefine the indications for UT but rather represents a technical evolution of an already established procedure.

From a surgical standpoint, robotic-assisted UT appears feasible and safe. Perioperative morbidity was limited, with no reported cases of uterine necrosis or irreversible ischemia, and complications were infrequent and manageable.

The consistent adoption of intra-abdominal cervical positioning combined with pharmacologic menstrual suppression across robotic cases reflects an important evolution from early laparoscopic experiences, in which cervical exteriorization was associated with higher rates of ischemic complications. This shift highlights how incremental technical refinements, rather than platform superiority alone, have contributed to improved procedural safety.

Functional outcomes following robotic-assisted UT are encouraging but remain preliminary. Preservation of ovarian endocrine function and resumption of menstruation were consistently reported in all evaluable patients (6/6) [24–28, 30, 31].

Only one reported case has provided information on attempted conception, resulting in a successful spontaneous pregnancy and live birth [25].

Oncologic outcomes across the robotic series were reassuring within the constraints of available follow-up. All patients completed planned oncologic treatment without delay or modification attributable to UT, and no recurrences directly related to the procedure were reported. These observations support the oncologic safety of robotic-assisted UT without compromising standard treatment protocol when performed in highly selected patients and integrated within a multidisciplinary treatment strategy.

### Comparison with historical experience

Interpretation of perioperative and functional outcomes following robotic-assisted UT should be contextualized within the previously published historical experience, which is largely, but not exclusively, laparoscopic. Interpretation of perioperative and functional outcomes following robotic-assisted UT should be contextualized within the previously published historical experiences, which is largely, but not exclusively, laparoscopic. Although robotic assistance offers technical advantages [33], there is currently no data demonstrating its clinical superiority over conventional laparoscopic techniques for uterine transposition. In the hands of experienced laparoscopic surgeons, these highly complex cases can be performed exceptionally well with the standard minimally invasive approach [13, 14]. Experienced laparoscopic surgeons can perform advanced pelvic and abdominopelvic dissections with excellent outcomes, and laparoscopy may be equivalent (or preferable) in expert hands.Furthermore, conventional laparoscopy often allows for smaller incision sizes compared to robotic ports, potentially translating into better cosmetic outcome, which should be considered during counseling of young patients. Finally, increased adoption of robotic platforms in pelvic surgery may reflect availability and institutional practice patterns rather than procedure-specific evidence; regardless of approach, structured training and concentration of cases in high-expertise centers remain essential.

In their comprehensive narrative review, Ribeiro et al. analyzed 18 UT cases reported up to 2023, the vast majority of which (16/18) were performed laparoscopically, with the inclusion of a single open surgery case and a single robotic-assisted case [13].

In the initial laparoscopic reports, cervical exteriorization was primarily adopted to allow continuous drainage of menstrual blood during pelvic radiotherapy, in the absence of systematic pharmacologic menstrual suppression. This strategy was intended to prevent hematometra and reduce the risk of infection during prolonged oncologic treatment. However, subsequent clinical experience demonstrated that exteriorization of the cervix was associated with a substantial risk of impaired cervical perfusion, late cervical ischemia, and stenosis, likely related to traction, kinking of residual vascular supply, and prolonged exposure of the cervical tissue to the external environment [14, 17, 18]. These complications prompted a progressive shift in surgical strategy. In our robotic series, routine preoperative pharmacologic menstrual suppression has allowed avoidance of cervical exteriorization, while maintaining effective menstrual control throughout radiotherapy.

Across this predominantly laparoscopic cohort, cervical ischemia emerged as the most frequently reported complication, occurring in approximately 28% of patients, and was mainly associated with early technical strategies involving cervical exteriorization through the umbilicus. In contrast, no cases of clinically significant cervical ischemia or uterine necrosis have been reported in the subsequent robotic-assisted series to date. Although these findings must be interpreted cautiously given the limited number of robotic cases and short follow-up, they likely reflect an evolution in surgical technique. Specifically, robotic series uniformly adopted intra-abdominal cervical positioning combined with systematic pharmacologic menstrual suppression.

When considering the laparoscopic experience, five patients attempted conception (33%), resulting in three patients (60%) achieving spontaneous pregnancies and three live births carried beyond 36 weeks gestation, with no reported miscarriages [17, 18]. However, the small number of patients attempting pregnancy and the limited duration of follow-up preclude definitive conclusions and comparison regarding fertility potential after UT.

### Limitations

Despite these encouraging observations, several limitations must be acknowledged.

The evidence is limited to isolated case reports, often reported as educational videos, with heterogeneous reporting of operative metrics and outcomes. This inconsistency limits meaningful comparison across cases and surgical approaches, as key perioperative endpoints (e.g., operative time, estimated, blood loss, length of stay, complication reporting) were variably captured and not uniformly defined. Moreover, postoperative monitoring and functional assessment lacked standardization: perfusion evaluation, ultrasound follow-up, and tubal patency testing were inconsistently performed and reported with non-uniform methodologies and timing, precluding any attempt to derive structured comparative inferences.

Follow-up remains short, particularly with respect to reproductive and oncologic endpoints. Additionally, overlap between laparoscopic and robotic reports within narrative reviews complicates aggregate analysis and underscores the need for standardized reporting frameworks.

Lastly, the possibility of publication bias must be acknowledged, as additional UT cases may have been performed but not reported in the published literature, potentially leading to an underestimation of the overall global experience.

### Implications for future research

Further insights are needed from prospective multicenter registries with standardized metrics, timing and outcome definitions for surgical, endocrine, reproductive, and oncologic outcomes.

Long-term follow-up is essential to determine not only pregnancy rates but also obstetric outcomes and uterine integrity over time.

Comparative analyses between laparoscopic and robotic approaches, while currently unfeasible, may become possible as experience accrues.

## Conclusion

Robotic-assisted UT represents a technically feasible refinement of an established fertility-preserving strategy in carefully selected patients requiring pelvic radiotherapy. While early outcomes suggest safety with preservation of endocrine and uterine function, the procedure should remain confined to specialized centers with multidisciplinary expertise until more robust evidence becomes available. Overall, robotic assistance appears to offer a promising technical evolution of UT, while current data underscore its still experimental nature and the need for further prospective investigation.

## Data Availability

No datasets were generated or analysed during the current study.
