# Correction: Robotic uterine transposition for fertility preservation in patients undergoing pelvic radiotherapy: a narrative review of surgical evolution, technical strategies, and emerging evidence

**DOI:** 10.1007/s11701-026-03356-y

**Published:** 2026-04-03

**Authors:** M. D’Indinosante, G. Parisi, Matteo Bruno, C. Innocenzi, M. Pavone, G. Chiloiro, R. Autorino, M. A. Gambacorta, I. Peters, G. Corrado, D. Querleu, F. Fanfani, A. Fagotti, N. Bizzarri

**Affiliations:** 1 Dipartimento Scienze della Salute della Donna, del Bambino e di Sanità Pubblica, Unità Operativa Complessa di Ginecologia Oncologica, Fondazione Policlinico Universitario A. Gemelli, IRCCS, Largo Agostino Gemelli, 8, Rome, 00136 Italy; 2 Università Cattolica del Sacro Cuore, Largo Francesco Vito, 1, Rome, 00168 Italy; 3 Department of Woman’s and Child Health and Public Health Sciences, Ovarian Cancer Unit, Fondazione Policlinico Universitario A. Gemelli IRCCS, Largo A. Gemelli 8, Rome, 00168 Italy; 4 Dipartimento di Diagnostica per Immagini, Radioterapia Oncologica ed Ematologia, Fondazione Policlinico Universitario ‘A. Gemelli’ IRCCS, Rome, Italy

**Correction: Journal of Robotic Surgery (2026) 20:332**

10.1007/s11701-026-03296-7

In the original version of the article, three authors were inadvertently omitted. The author list which previously appeared as

M. D’Indinosante · G. Parisi · Matteo Bruno · C. Innocenzi · M. Pavone · I. Peters · G. Corrado · D. Querleu · F. Fanfani · A. Fagotti · N. Bizzarri

But should have been

M. D’Indinosante · G. Parisi · Matteo Bruno · C. Innocenzi · M. Pavone · G. Chiloiro · R. Autorino · M. A. Gambacorta · I. Peters · G. Corrado · D. Querleu · F. Fanfani · A. Fagotti · N. Bizzarri

